# Multi-phasic gene profiling using candidate gene approach predict the capacity of specific antibody production and maintenance following COVID-19 vaccination in Japanese population

**DOI:** 10.3389/fimmu.2023.1217206

**Published:** 2023-07-26

**Authors:** Yuki Takemoto, Naoki Tanimine, Hisaaki Yoshinaka, Yuka Tanaka, Toshiro Takafuta, Aya Sugiyama, Junko Tanaka, Hideki Ohdan

**Affiliations:** ^1^ Department of Gastroenterological and Transplant Surgery, Graduate School of Biomedical and Health Sciences, Hiroshima University, Hiroshima, Japan; ^2^ Department of Internal Medicine, Hiroshima City Funairi Citizens Hospital, Hiroshima, Japan; ^3^ Department of Epidemiology, Infectious Disease Control and Prevention, Graduate School of Biomedical and Health Sciences, Hiroshima University, Hiroshima, Japan

**Keywords:** candidate gene approach, gene polymorphism, COVID-19, vaccine, humoral immunity, specific antibody

## Abstract

**Background:**

Vaccination against severe acute respiratory syndrome coronavirus type 2 is highly effective in preventing infection and reducing the severity of coronavirus disease (COVID-19). However, acquired humoral immunity wanes within six months. Focusing on the different tempo of acquisition and attenuation of specific antibody titers in individuals, we investigated the impact of genetic polymorphisms on antibody production after COVID-19 vaccination.

**Methods:**

In total 236 healthcare workers from a Japanese municipal hospital, who received two doses of the vaccine were recruited. We employed a candidate gene approach to identify the target genetic polymorphisms affecting antibody production after vaccination. DNA samples from the study populations were genotyped for 33 polymorphisms in 15 distinct candidate genes encoding proteins involved in antigen-presenting cell activation, T cell activation, T-B interaction, and B cell survival. We measured total anti-SARS-Cov2 spike IgG antibody titers and analyzed the association with genetic polymorphisms at several time points after vaccination using an unbiased statistical method, and stepwise logistic regression following multivariate regression.

**Results:**

Significant associations were observed between seven SNPs in *NLRP3, OAS1, IL12B, CTLA4*, and *IL4*, and antibody titers at 3 weeks after the first vaccination as an initial response. Six SNPs in *NLRP3, TNF, OAS1, IL12B*, and *CTLA4* were associated with high responders with serum antibody titer > 4000 BAU/ml as boosting effect at 3 weeks after the second vaccination. Analysis of long-term maintenance showed the significance of the three SNPs in *IL12B, IL7R*, and *MIF* for the maintenance of antibody titers and that in *BAFF* for attenuation of neutralizing antibodies. Finally, we proposed a predictive model composed of gene profiles to identify the individuals with rapid antibody attenuation by receiver operating characteristic (ROC) analysis (area under the curve (AUC)= 0.76, sensitivity = 82.5%, specificity=67.8%).

**Conclusions:**

The candidate gene approach successfully showed shifting responsible gene profiles and initial and boosting effect mainly related to the priming phase into antibody maintenance including B cell survival, which traces the phase of immune reactions. These gene profiles provide valuable information for further investigation of humoral immunity against COVID-19 and for building a strategy for personalized vaccine schedules.

## 1 Introduction

The severe acute respiratory syndrome coronavirus type 2 (SARS-CoV-2) pandemic has caused a major global health crisis (1, 2), and vaccines have been developed worldwide (3). Several randomized trials have shown that mRNA vaccines are highly effective in preventing infection and reducing the severity of COVID-19 (4–6). Almost of cases could obtain sufficient amounts of specific antibody production immediately after the vaccination, however, a marked decrease in serum antibody titers was observed at approximately 6 months after the second vaccination (7–9). Along with a decrease in antibody titers, waning immunity against COVID-19 infection preventive effect has been reported, especially in males and older individuals (8).

Host factors such as age, sex, comorbidities, and genetic polymorphisms have been shown to influence individual immune status including acquired immunity after vaccination (10, 11). The effect of host-side genetic factors on vaccine response has been demonstrated in conventional vaccines, such as hepatitis B virus, pneumococcus pneumoniae, and measles (12–17). However, it is unclear whether an association exists between genetic polymorphisms and responses to vaccination against COVID-19.

In the present study, we employed a candidate gene approach, which selects a series of target genes based on the rationale of biological response or mechanism (18), to investigate the impact of genetic polymorphisms on antibody production after COVID-19 vaccination. Furthermore, we analyzed the effects of the gene profile in an immunological network using an unbiased statistical method, a stepwise regression method, and identified genetic factors as predictors of high response and antibody maintenance.

## 2 Materials and methods

### 2.1 Study participants

Our study population was similar to the previous report (19). A total of 236 Japanese healthcare workers working at Funairi Citizens Hospital in Japan who received their first dose of the vaccine between March and May 2021 participated in the study. All the participants received two doses of BNT162b2 (Pfizer/Biotech). The vaccinations were administered at the intervals specified in the protocol, that is the second dose was administered three weeks after the first dose. Ultimately, 213 participants with no missing data were included in the analysis, including 35 males (16.4%) and 178 females (83.6%). The age distribution was as follows: 20–29 years: n=29 (13.6%), 30–39 years: n=54 (25.4%), 40–49 years: n=58 (27.2%), 50–59 years: n=45(21.1%), and ≥60 years: n=27 (12.7%).

This study was approved by the Ethics Committee for Human Genome Analysis at Hiroshima University (Hi-258). Written informed consent was obtained from all the participants.

### 2.2 Measurement of anti-SARS-Cov-2 spike IgG antibodies

Blood samples were collected three weeks after the first vaccination (just before the second vaccination), three weeks after the second vaccination, and five months after the second vaccination. Total anti-SARS-Cov2 spike IgG antibodies were quantitatively measured using the VITROS SARS-Cov-2 S1 Quant IgG antibody reagent (CLEIA, Ortho Clinical Diagnostics). Quantitative values were determined using the WHO standard binding antibody unit/ml (BAU/ml) (20). The upper limit of quantification was 4000 BAU/ml. Participants with specific N-antibodies detected by the Elecsys^®^Anti-SARS-CoV-2 (ECLIA, Roche Diagnostics) were defined as previously infected and excluded from the analysis.

### 2.3 Candidate gene polymorphisms

**Figure 1:**
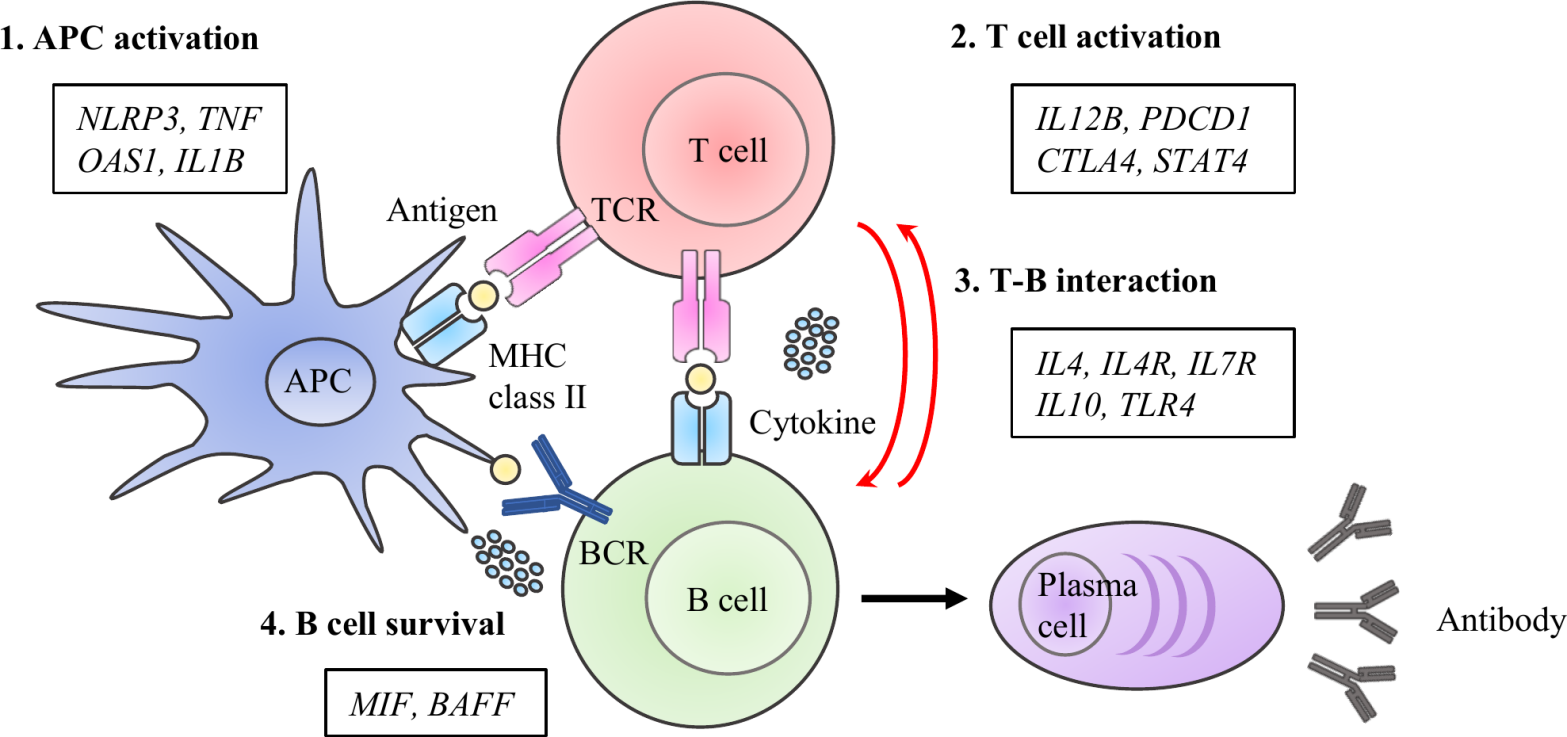
Immuno-network for antibody production after COVID-19 vaccine. We selected 15 molecules and 33 SNPs categorized into priming- associated APC and T cell activation, B cell activation with T -B interactions, and B cell survival, which are key immunological steps for enhancing to enhance specific antibody production after antigen exposure.

DNA samples from the study subjects were genotyped for 33 functional polymorphisms in 15 candidate genes. The candidate genes were selected on pathophysiological hypotheses based on the best evidence from studies published under the keywords, “vaccine,” “immune response,” and “antibody production.” Single nucleotide polymorphisms (SNPs) in genes encoding functional molecules involved in the immune responses that lead to the establishment of vaccine-induced acquired immunity were investigated. These processes can be categorized into the following four phases: 1) antigen-presenting cell (APC) activation, 2) T cell activation, 3) T cell and B cell (T-B) interaction, and 4) B cell survival (**Figure 1**). If there were more than four candidate polymorphisms in a gene, selection was based on the number of publications in the single nucleotide polymorphism (SNP) database of the National Center for Biotechnology Information (21, 22). The list of each SNPs and its allele frequency is presented in **Table 1** (12–17, 23–51).

**Table 1:** Description of candidate genes related to humoral immunity.

Gene	SNP site	Gene locus (GRCH38.p13)	Location	Major allele	Minor allele	Minor allele frequency*	References
APC activation							
*NLRP3*	rs4612666	chr1:247435768	Intron	C	T	0.38 (0.45)	(23)
	rs10925027	chr1:247449260	500B Downstream	T	C	0.56 (0.47)	(24)
*TNF*	rs1799724	chr6:31574705	2KB Upstream	C	T	0.21 (0.13)	(13, 25)
	rs1799964	chr6:31574531	2KB Upstream	T	C	0.17 (0.20)	(26, 27)
*OAS1*	rs1131454	chr12:112911065	Exon	A	G	0.38 (0.41)	(28, 29)
*IL1B*	rs1143627	chr2:112836810	5′ UTR	A	G	0.48 (0.48)	(28, 30)
T cell activation							
*IL12B*	rs6887695	chr5:159395637	60KB upstream	G	C	0.46 (0.42)	(31)
	rs3212227	chr5:159315942	3′ UTR	G	T	0.50 (0.49)	(12, 32)
	rs2546893	chr5:159328952	Intron	G	A	0.46 (0.45)	(17)
*PDCD1*	rs36084323	chr2:241859444	2KB Upstream	C	T	0.49 (0.47)	(33)
	rs2227981	chr2:241851121	Exon	G	A	0.28 (0.27)	(34)
	rs10204525	chr2:241850169	3′ UTR	T	C	0.27 (0.34)	(35)
*CTLA4*	rs231775	chr2:203867991	Exon	G	A	0.39 (0.36)	(36)
	rs3087243	chr2:203874196	500B Downstream	G	A	0.28 (0.26)	(37)
*STAT4*	rs7574865	chr2:191099907	Intron	G	T	0.33 (0.35)	(38, 39)
	rs7572482	chr2:191150346	Intron	A	G	0.51 (0.39)	(40)
	rs3821236	chr2:191038032	Intron	G	A	0.43 (0.41)	(26)
T-B interaction							
*IL4*	rs2243250	chr5:132673462	2KB Upstream	T	C	0.31 (0.22)	(12, 14–16)
	rs2070874	chr5:132674018	5′ UTR	T	C	0.31 (0.22)	(15, 16)
	rs2227284	chr5:132677033	Intron	T	G	0.25 (0.15)	(14)
*IL4R*	rs1805010	chr16:27344882	Exon	G	A	0.42 (0.48)	(12, 16)
	rs1801275	chr16:27363079	Exon	A	G	0.13 (0.17)	(16)
*IL7R*	rs6897932	chr5:35874473	Exon	C	T	0.17 (0.20)	(27, 32)
	rs1494555	chr5:35871088	Exon	G	A	0.50 (0.48)	(41)
	rs1494558	chr5:35860966	Exon	T	C	0.47 (0.43)	(41)
*IL10*	rs1800872	chr1:206773062	2KB Upstream	T	G	0.28 (0.32)	(42, 43)
	rs1800871	chr1:206773289	2KB Upstream	A	G	0.32 (0.32)	(16, 42)
*TLR4*	rs10759932	chr9:117702866	2KB Upstream	T	C	0.23 (0.24)	(44)
	rs1927914	chr9:117702447	2KB Upstream	A	G	0.35 (0.37)	(45)
B cell survival							
*BAFF*	rs9514828	chr13:108269025	Non-Coding Transcript	C	T	0.44 (0.36)	(46, 47)
	rs12583006	chr13:108285104	Intron	T	A	0.47 (0.45)	(46, 48, 49)
*MIF*	rs755622	chr22:23894205	2KB Upstream	G	C	0.21 (0.20)	(50)
	rs1007888	chr22:23898914	Non-Coding Transcript	C	T	0.47 (0.46)	(51)

APC, Antigen-presenting cell; SNP, Single nucleotide polymorphism; T-B interaction, T and B cell interaction

* Data of East Asian population from Genome 1000 date base (Our samples).

### 2.4 Genotyping

Genetic analyses were performed as previously described (28). Briefly, blood samples from the study subjects were collected in EDTA tubes and assigned a blinded unique identification number. Genomic DNA was extracted from whole blood samples using a QIA Cube (QIAGEN, Hilden, Germany). SNP genotyping was performed using TaqMan SNP Genotyping Assays (Thermo Fisher Scientific, MA) according to the manufacturer’s protocol. Two allele specific TaqMan probes containing different fluorescent dyes and polymerase chain reaction (PCR) primer pairs were used to detect specific SNP targets. Quantitative PCR was performed using a Rotor-GeneQ (QIAGEN). We described the zygosity of the A>G SNP as AA or GG and AG in the case of homozygosity and heterozygosity, respectively. Genotypes were analyzed in either a recessive or dominant model, which was selected as the more impactful model based on the results of previous studies or our data set.

### 2.5 Effects of gene profiles on antibody titers after vaccination

We analyzed the association between antibody titers and genetic polymorphisms at 3 weeks after the first vaccination, 3 weeks after the second vaccination, and 5 months after the second vaccination, which was analyzed as initial response, boosting effect, and maintenance ability of antibodies, respectively.

Analyzing the impact of genetic profile for boosting effect, we divided subjects into 2 groups: the individuals with a serum antibody level ≥4000 BAU/ml (upper limit of measurement) defined as high responders and those with <4000 BAU/ml defined as low responders at 3 weeks after the second vaccination.

To account for the level of antibody titer at boosting effect for following maintenance ability, subjects were categorized into three groups by the antibody titers at 3 weeks after the second vaccination (≥4000 BAU/ml, ≥1825 BAU/ml, and <4000 BAU/mL, or <1825 BAU/ml). The median antibody titer at 3 weeks after the second vaccination was 1825 BAU/ml, excluding high responders.

To analyze of the maintenance of protective antibodies, we focused on the neutralizing antibodies. Previously, our collaborators have reported a proportional relationship between the total IgG levels of anti-S protein antibodies and neutralizing activity (19). The predictive formula was as follows: neutralizing inhibitory activity (%) = 48.2 × log (total IgG level of anti-S protein BAU/ml) - 53.5. The negative threshold for neutralizing activity was evaluated using a surrogate virus neutralization test, which showed 30% inhibition compared with negative sera (52). A total IgG level of 54 BAU/ml, which was expected to maintain 30% neutralizing activity, was calculated using a predictive formula. We analyzed the predictive gene profiles below the negative threshold.

### 2.6 Statistical analysis

All statistical analyses were performed using JMP Pro statistical software package 15.0.0 (SAS Institute, Cary, NC, USA). We used multivariate multiple regression and multivariate logistic regression analyses to identify the factors that were significantly associated with antibody production and maintenance. We used stepwise logistic regression with forward-backward elimination to determine the best combination of variables. The accuracy of the predictive model for high responders and maintenance was assessed using the area under the receiver operating characteristic (ROC) curve.

## 3 Results

### 3.1 Polymorphic gene profiles affect the initial response after the first COVID-19 vaccination

First, we analyzed the demographic and genetic variables associated with specific antibody production in the initial response phase after the first COVID-19 vaccination. Ten of the 33 candidate SNPs were defined as potential factors by stepwise regression, and seven of the 10 SNPs and young age (<60 years) were significantly associated with the absolute titer of anti-SARS-CoV-2 spike IgG in the initial response phase (**Table 2**). The statistically significant seven SNPs were within genes encoding immune function molecules belonging to multiple phases, i.e., rs4612666 (*NLRP3*), rs10925027 (*NLRP3*), and rs1131454 (*OAS1*) upon APC activation phase; rs3212227 (*IL12B*), rs2546893 (*IL12B*), and rs3087243 (*CTLA4*) on T cell activation phase, and rs2227284 (*IL4*) on T-B interaction phase. Thus, SNPs in genes encoding molecules responsible for functions in immunological processes from the priming to B cell activation phases were important predictors in favor of antibodies in response to the initial vaccine, whereas SNPs encoding molecules belonging to the B cell survival phase showed no significant association.

**Table 2:** Demographic and genetic valuables associated with higher antibody titer in an initial response phase.

Demographics		Variables	n (%)	IgG titer: median (IQR)	p value
Age		< 60	186 (87.3)	308.0 (120.8-500.5)	<0.001
		≥ 60	27 (12.7)	74.7 (60.9-122.0)	
Sex		Male	35 (16.4)	156.0 (65.6-385.0)	0.247
		Female	178 (83.6)	276.0 (111.0-722.6)	
Genetic variables	SNP sites	Genotype	n (%)	IgG titer: median (IQR)	p value
APC activation					
*NLRP3*	rs4612666* (C>T)	CC	78 (36.6)	175.5 (89.6 - 400.3)	0.009
		CT+TT	135 (63.4)	307.0 (121.0-506.0)	
	rs10925027* (T>C)	TT+TC	175 (82.2)	220.0 (96.0-445.0)	0.008
		CC	38 (17.8)	374.5 (143.0-531.0)	
*OAS1*	rs1131454* (A>G)	AA+AG	180 (84.5)	235.5 (97.0-444.8)	0.047
		GG	33 (15.5)	378.0 (132.5-515.5)	
T cell activation					
*IL12B*	rs3212227* (G>T)	GG+GT	162 (76.1)	255.0 (98.7-474.8)	0.005
		TT	51 (23.9)	225.0 (114.0-481.0)	
	rs2546893* (G>A)	GG+GA	167 (78.4)	241.0 (92.4-474.0)	0.020
		AA	46 (21.6)	323.0 (121.0-488.8)	
*CTLA4*	rs3087243* (G>A)	GG	24 (11.3)	168.5 (88.5-385.3)	0.011
		GA+AA	189 (88.7)	272.0 (109.0-482.5)	
T-B interaction					
*IL4*	rs2227284* (T>G)	TT	121 (56.8)	296.0 (109.0-509.5)	0.003
		TG+GG	92 (43.2)	238.0 (88.4-413.0)	
*IL7R*	rs6897932* (C>T)	CC	121 (56.8)	236.0 (96.0-478.5)	0.147
		CT+TT	92 (43.2)	268.0 (118.5-472.8)	
B cell survival					
*BAFF*	rs12583006* (T>A)	TT+TA	171 (80.3)	236.0 (99.5-476.0)	0.203
		AA	42 (19.7)	297.5 (102.8-513.3)	
*MIF*	rs755622* (G>C)	GG	124 (58.2)	255.0 (105.8-510.5)	0.248
		GC+CC	89 (41.8)	241.0 (95.8-418.0)	

IQR, Interquartile range; SNP, Single nucleotide polymorphism; T-B interaction, T cell and B cell interaction

*Ten of 33 SNPs were selected as potential predictive variables using stepwise regression.

### 3.2 The boosting response is also associated with priming phase gene profiles

Three weeks after the second vaccination, we observed 53 high responders with anti-SARS-CoV-2 spike IgG at more than 4000 BAU/ml. Ten SNPs in the 33 candidate genes were defined as potential factors using stepwise regression, and six SNPs and young age were significantly associated with high responders (**Table 3**). Four of the six genes related to high responders overlapped with those related to initial response, i.e., rs4612666 (*NLRP3*) and rs1131454 (*OAS1*) on APC activation phase, and rs3212227 and rs2546893 (*IL12B*) on T cell activation phase.

**Table 3:** Demographic and genetic valuables for predicting high responders in a boost response phase.

Demographics		Variables	OR	95%CI of OR		p value
				Lower	Upper	
Age		< 60	4.12	1.06	16.03	0.041
Sex		Male	0.31	0.10	1.03	0.056
Genetic variables	SNP sites	Genotype	OR	95%CI of OR		p value
				Lower	Upper	
APC activation						
*NLRP3*	rs4612666* (C>T)	CC	0.38	0.17	0.86	0.019
	rs10925027* (T>C)	CC	1.99	0.84	4.76	0.119
*TNF*	rs1799724* (C>T)	CC	0.47	0.23	0.99	0.047
	rs1799964* (T>C)	CC	5.00	0.72	34.90	0.105
*OAS1*	rs1131454* (A>G)	GG	4.09	1.65	10.17	0.002
*IL1B*	rs1143627* (A>G)	GG	2.01	0.90	4.49	0.088
T cell activation						
*IL12B*	rs3212227* (G>T)	TT	5.76	1.31	25.28	0.020
	rs2546893* (G>A)	AA	0.21	0.04	0.10	0.049
*CTLA4*	rs231775* (G>A)	GG	0.38	0.18	0.82	0.014
	rs3087243* (G>A)	AA	0.41	0.12	1.38	0.150

APC, Antigen-presenting cell; CI, Confidence interval; OR, Odds ratio; SNP, Single nucleotide polymorphism

*Ten of 33 SNPs were selected as potential predictive variables using stepwise regression.

**Figure 2:**
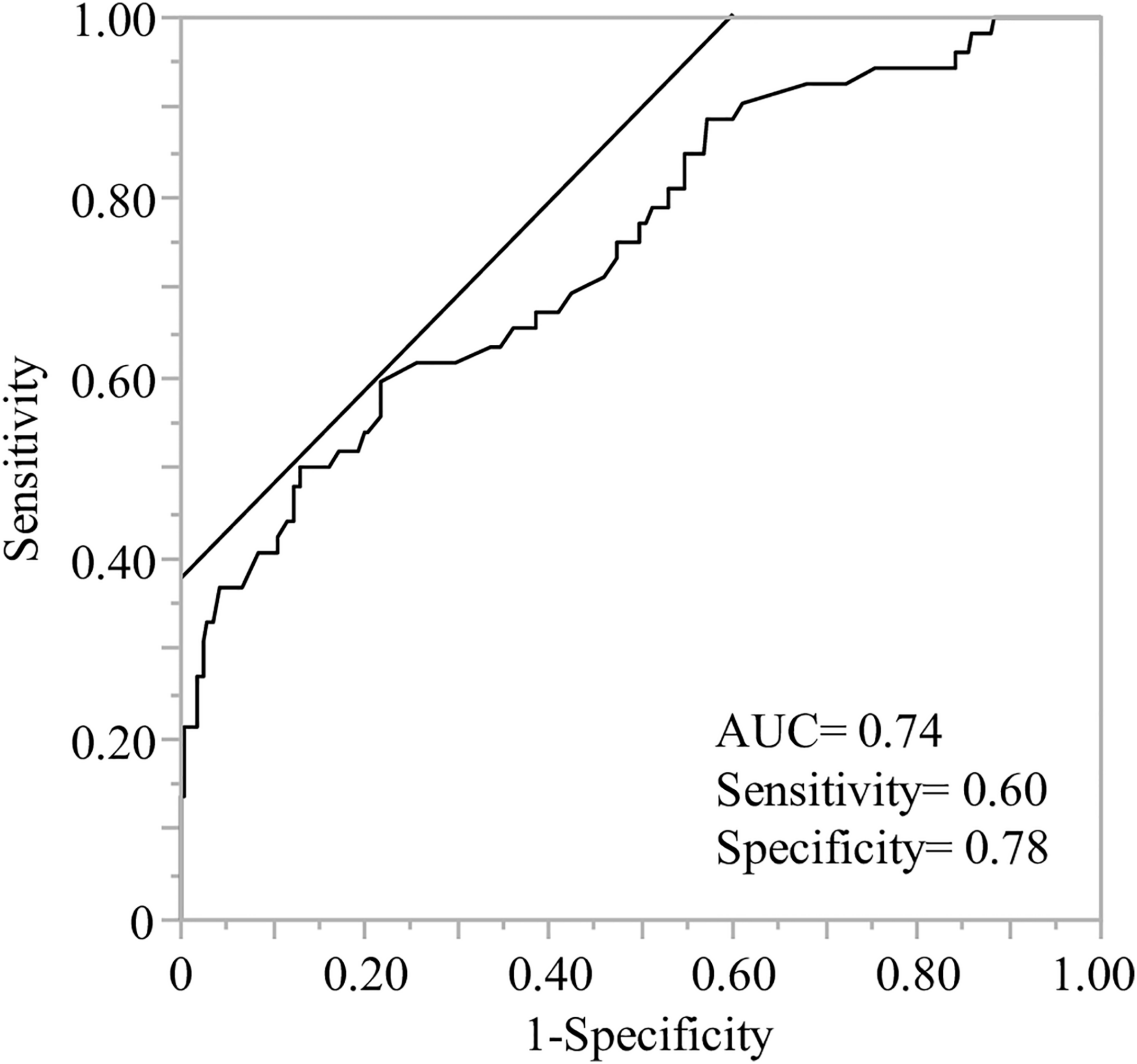
Receiver operating characteristic (ROC) analysis of predictive models to identify the high responders after the second vaccination. ROC curve analysis was performed using the indicated age, sex, and genetic factors to calculate the predictive value, area under the curve (AUC), sensitivity, and specificity. The demographics and ten genes indicated age less than 60, sex (female) and rs4612666 and rs10925027 (*NLRP3*), rs1799724 and rs1799964 (*TNF*), rs1131454 (*OAS1)*, rs1143627 (*IL1B*), rs3212227 and rs2546893 (*IL12B*), rs231775 and rs3087243 (*CTLA4*), respectively (details are described in **Table 3**).

We proposed a model for predicting high responders using polygenetic factors and age with a receiver operating characteristic (ROC) curve analysis (area under the curve (AUC) =0.74, **Figure 2**). The model equation was as follows; prediction score (P) = 1/(1+e-x) x = -1.09 + 0.71×(age: -1 if 60 years, 1 if under 60)+ 0.58×(sex: -1 if male, 1 if female)+ 0.48×(*NLRP3* rs4612666: -1 if CC, 1 if T carrier)+ 0.35×(*NLRP3* rs10925027: -1 if T carrier, 1 if CC)+ 0.37×(*TNF* rs1799724: -1 if CC, 1 if T carrier)+ 0.80×(*TNF* rs1799964: -1 if T carrier, 1 if CC)+ 0.70×(*OAS1* rs1131454: -1 if A carrier, 1 if GG)+ 0.35×(*IL1B* rs1143627: -1 if A carrier, 1 if GG)+ 0.88×(*IL12B* rs3212227: -1 if G carrier, 1 if TT)+ 0.77×(*IL12B* rs2546893: -1 if AA, 1 if G carrier)+ 0.48×(*CTLA4* rs231775: -1 if GG, 1 if A carrier)+ 0.44×(*CTLA4* rs3087243: -1 if AA, 1 if G carrier).

### 3.3 Polymorphic gene profile for long-term maintenance of specific antibody after vaccination

By analyzing the effect of gene profiles on the ability to maintain antibody titers 5 months after the second vaccination, seven SNPs were defined as potential factors by stepwise regression. Three of the 7 SNPs, rs3212227 (*IL12B*) in the T cell activation phase, rs1494555 (*IL7R*) in the T-B interaction phase, and rs1007888 (*MIF*) in the B cell survival phase, were significantly associated with the maintenance of high titers of anti-SARS-CoV-2 spike IgG (**Table 4**).

**Table 4:** Demographic and genetic variables associated with higher antibody titer in a long-term maintenance phase.

Demographics		Variables	n (%)	IgG titer: median (IQR)	p value
Antibody titer after 2nd vaccination (BAU/ml)		≥ 1825	133 (62.4)	214.0 (169.0-325.0)	<0.001
		< 1825	80 (37.6)	83.4 (51.7-143.2)	
Genetic variables	SNP sites	Genotype	n (%)	IgG titer: median (IQR)	p value
T cell activation					
*IL12B*	rs3212227* (G>T)	GG+GT	162 (76.1)	171.0 (86.9-236.0)	0.008
		TT	51 (23.9)	171.0 (105.0-518.4)	
*PDCD1*	rs2227981* (G>A)	GA+AA	105 (49.3)	162.0 (82.3-241.0)	0.209
		GG	108 (50.7)	177.0 (112.3-258.0)	
T-B interaction					
*IL4R*	rs1801275* (A>G)	AA	160 (75.1)	170.5 (86.7-254.8)	0.098
		AG+GG	53 (24.9)	172.0 (105.0-251.0)	
*IL7R*	rs1494555* (G>A)	GG	49 (23.0)	172.0 (84.0-245.0)	0.019
		GA+AA	164 (77.0)	170.5 (94.3-254.0)	
*TLR4*	rs10759932* (T>C)	TT+TC	201 (94.4)	172.0 (94.9-255.5)	0.236
		CC	12 (5.6)	114.0 (70.4-191.5)	
B cell survival					
*BAFF*	rs9514828* (C>T)	CC	64 (30.0)	177.0 (94.3-280.8)	0.137
		CT+TT	149 (70.0)	164.0 (86.8-249.5)	
*MIF*	rs1007888* (C>T)	CC	58 (27.2)	176.0 (110.8-276.3)	0.031
		CT+TT	155 (72.8)	164.0 (87.0-248.0)	

IQR, Interquartile range; SNP, Single nucleotide polymorphism; T-B interaction, T and B cell interaction

*Seven of 33 SNPs were selected as potential predictive variables using stepwise regression.

**Figure 3:**
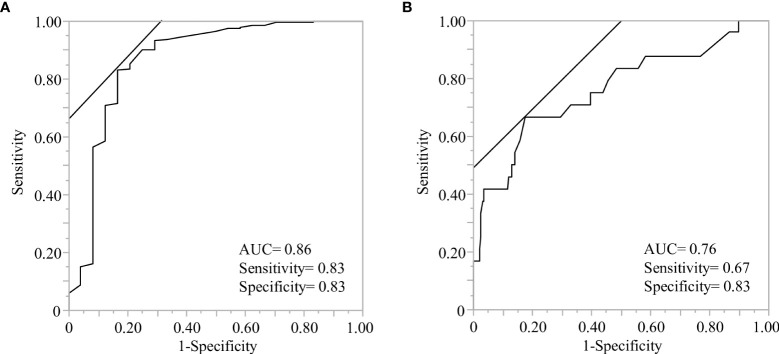
Receiver operating characteristic (ROC) analysis of predictive models to identify the individuals with rapid antibody attenuation. ROC curve analysis was performed using the indicated genetic factors with or without antibody titers, after the second vaccination. **(A)** The model consisted of the indicated genetic factors and antibody titers after the second vaccination. The demographics and six genes indicated gender(male), antibody titer after the second vaccination less than 1825 BAU/ml, rs10925027 (*NLRP3*), rs1143627 (*IL1B*), rs2546893 (*IL12B*), rs1805010 (*IL4R*), rs1800872 (*IL10*), and rs12583006 (*BAFF*) (details are described in **Table 5**). **(B)** Model consisting of demographics and indicated genetic factors without antibody titer information. The demographics and seven genes indicated age less than 60 years, sex (male), rs4612666 and rs10925027 (*NLRP3*), rs6887695 (*IL12B*), rs7572482 (*STAT4*), rs1805010 (*IL4R*), rs1494555 (*IL7R*), and rs12583006 (*BAFF*).

One of the current topics in COVID-19 prevention is the breakthrough infection after vaccination. It has been reported that the neutralizing potency, which is proportional to anti-SARS-CoV-2 spike receptor-binding domain IgG levels, is key to preventing COVID-19 infection (53, 54). We evaluated predictive factors for the individuals whose anti-SARS-CoV-2 spike IgG titer dropped below the negative threshold (details in Material and method) and found that rs12583006 (*BAFF*) was exclusively identified as a genetic risk factor for rapid attenuation of anti-SARS-CoV-2 IgG (aOR 0.23, 95%CI 0.07-0.80) (**Table 5**). We proposed a predictive model to identify individuals with rapid anti-SARS-CoV-2 IgG attenuation using ROC curve analysis with or without antibody titer information (AUC=0.86, and 0.76, respectively, **Figures 3A, B**). Although the prediction model with anti-SARS-CoV-2 IgG titer information was more accurate, the model consisting of the gene profile alone without antibody titer remained informative. The model equation was as follows; predicted score (P)=1/(1+e-x) x=-1.16 + 0.50×(sex: 1 if male, -1 if female)+ 0.42×(age: 1 if 60 years, -1 if under 60 years)+ 0.38×(*NLRP3* rs4612666: -1 if T carrier, 1 if CC)+ 0.91×(*NLRP3* rs10925027: -1 if CC, 1 if T carrier)+ 0.40×(*IL12B* rs6887695: -1 if G carrier, 1 if CC)+ 0.47×(*STAT4* rs7572482: -1 if A carrier, 1 if GG)+ 0.51×(*IL4R* rs1805010: -1 if A carrier, 1 if GG)+ 0.40×(*IL7R* rs1494555: -1 if A carrier, 1 if GG)+ 0.50×(*BAFF* rs12583006: -1 if T carrier, 1 if AA), (AUC=0.76, **Figure 3B**).

**Table 5:** Demographic and genetic variables associated with rapid neutralizing antibody attenuation.

Demographics		Variables	OR	95%CI of OR		p value
				Lower	Upper	
Sex		Male	0.38	0.11	1.31	0.127
Antibody titer after 2nd vaccination (BAU/ml)		< 1825	23.67	6.03	92.90	<0.001
Genetic variables	SNP sites	Genotype	OR	95%CI of OR		p value
				Lower	Upper	
APC activation						
*NLRP3*	rs10925027* (T>C)	CC	8.64	0.99	75.33	0.051
*IL1B*	rs1143627* (A>G)	GG	3.15	0.83	11.97	0.091
T cell activation						
*IL12B*	rs2546893* (G>A)	AA	2.64	0.70	9.88	0.149
T-B interaction						
*IL4R*	rs1805010* (A>G)	GG	0.52	0.18	1.49	0.222
*IL10*	rs1800872* (T>G)	TT	2.41	0.79	7.37	0.121
B cell survival						
*BAFF*	rs12583006* (T>A)	AA	0.23	0.07	0.80	0.021

APC, Antigen-presenting cell; CI, Confidence interval; OR, Odds ratio; SNP, Single nucleotide polymorphism; T-B interaction, T cell and B cell interaction

*Six of 33 SNPs were selected as potential predictive variables using stepwise regression.

## 4 Discussion

In this study, we investigated the effects of host genetic factors on the production and maintenance of specific antibodies after COVID-19 vaccination. Currently, the entire genome can be examined using comprehensive strategies such as genome-wide association studies (GWAS). Although the GWAS has the advantage of identifying novel variant-associated lesions and covering rare variants without any bias, it requires the large number of participants and amount of expense to carry out a study (18, 55). In a previous study, we employed a candidate gene approach to identify genetic factors associated with the severity of COVID-19 infection (28). Our study with a relatively small cohort successfully identified *OAS1* (*rs1311454*) as a risk factor for severe infection, consistent with the results of a large GWAS (56). We applied this approach as an efficient way, to analyze the association between specific antibody production after COVID-19 vaccination and host immune-associated gene polymorphisms. This is the first report to investigate the impact of genetic polymorphisms for COVID-19 vaccine response in the candidate gene approach.

Based on a series of immune responses, we selected 15 molecules and 33 SNPs categorized as priming associated APC and T cell activation, B cell activation with T-B interaction, and B cell survival, which are key immunological steps in enhancing specific antibody production after antigen exposure (11, 57, 58). To understand the immune response as a multi-directional interacting network, genetic polymorphisms were analyzed using unbiased statistical methods such as stepwise regression. Genetic analyses have revealed gene profiles that reflect the immune response after vaccination to produce and maintain a specific antibody against the SARS-CoV-2 spike receptor-binding domain. Antigen exposure triggers a series of responses in which APCs present antigens to T cells and antigen presented T cells activate B cells by activating molecules and cytokines resulting in the generation of memory B cells in a germinal center reaction (T cell-dependent pathway) (59). Grifoni et al. recently reported the significance of T cell responses to SARS-CoV-2 in specific antibody production by showing that spike-specific CD4+ T cell responses correlated with the magnitude of specific IgG titers in recovered COVID-19 patients (60). Simultaneously, APCs promote B cell activation and differentiation, such as class-switching recombination, in a T cell-independent manner (61, 62). In line with these mechanistic steps of B cell activation, we observed that the initial response and boosting effect were significantly associated with gene profiles of APC activation, T cell activation, and T-B interactions. Although some B cells differentiate into plasma cells to establish long-term humoral immunity after activation, several reports have suggested that current COVID-19 vaccines do not induce the differentiation into long-lived plasma cells, since the kinetics of antibody titers show a decrease in the following several months without exception in immunologically normal individuals (7–9). Therefore, antibody maintenance after COVID-19 vaccination depend on B cell survival. The maturation and survival of memory B cells depend on B cell receptor signaling and several cytokines such as BAFF and MIF (63, 64). Our data showed that the tempo of IgG decline after the second vaccination varied among individuals, and B cell survival-related genes affected the attenuation difference along with T cell activation and T-B interaction related genes. Thus, the transition of the responsible gene profile from the initial/boosting effects to antibody maintenance reflect the phase transition of immune responses, supporting the validity of the candidate gene approach employed in this study.

Among the gene profiles demonstrated in this study, the SNPs of NLRP3 and IL12B were consistently defined as potential factors for specific antibody production and maintenance after COVID-19 vaccination. These molecules and the SNPs in the genes encoding them may be key elements for further investigations into efficient vaccine development. Emerging reports have suggested that the cellular immunity induced by the COVID-19 vaccine may play an important role in protection against COVID-19 (65). The impact of genetic profiles on cellular immunity will be of great interest in future studies.

*OAS1*, a gene polymorphism in rs1131454 reported as a risk factor for severe COVID-19 infection (28, 56), is induced by IFN-γ and directly suppresses viral replication through viral RNA degradation (66). A recent report showed that OAS1 gain-of-function variants cause exogenous RNA-independent immunodeficiency disease through RNase L-mediated RNA cleavage leading to transcriptomic alterations, translational arrest, dysfunction and apoptosis of monocytes, macrophages, and B-cells (67). Because the mRNA vaccine itself induces type I IFN expression through MDA5 signaling (68), we included rs1131454 (*OAS1*) as a target gene in this study. We found that homozygosity of the minor allele in rs1131454 (*OAS1*) was associated with a high response to antibody production after vaccination, consistent with the protective effect against B cell/APC dysfunction through cellular RNA -cleavage.

Finally, our predictive model based on genetic polymorphisms could be useful for predicting individual vaccine efficacy and building a strategy for personalized vaccination schedules (**Figures 3A, B**). A third COVID-19 vaccine dose was recommended based on the reduction in vaccine effectiveness over time. One report showed that the protective effectiveness calculated using the Cox hazard model decreased from 88% at 1 month after the second vaccination to 47% after 5–6 months (69). However, little information is available on the development of appropriate vaccination schedules for individuals. Although routine measurement of antibody titers could be useful for monitoring immune status and vaccine efficacy, repeated measurements with routine follow-ups are inefficient in identifying declining antibody titers. Genetic profiling with basic demographics provides information to distinguish the potential for an early decline in antibody titers. Predictive models composed solely of genetic polymorphisms can provide useful information in clinical practice (**Figure 3B**).

This study had several limitations. First, we cannot discard the potential selection bias, as the enrolled population was female -dominant and consisted of a single Japanese race. Second, the modest sample size may have limited the power of the study. We believe that an appropriate methodology, such as the candidate gene approach can reveal the valuable effects of gene polymorphisms even with a modest sample size. Further studies involving other cohorts are required to validate our findings.

## 5 Conclusions

We revealed that the kinetics of anti-SARS-CoV-2 IgG titers after COVID-19 vaccination were associated with a rationalized multi-phasic gene profile, rather than physical senescence. These gene profiles provide valuable information for further investigations of humoral immunity against COVID-19 and for building a strategy for personalized vaccine schedules.

## Data availability statement

The original contributions presented in the study are included in the article/supplementary materials. Further inquiries can be directed to the corresponding authors.

## Ethics statement

The studies involving human participants were reviewed and approved by The Ethics Committee for Human Genome Analysis at Hiroshima University (Hi-258). The participants provided their written informed consent to participate in this study.

## Author contributions

YTak participated in acquisition, analysis and interpretation of data, and drafting of the manuscript. NT participated in study design, analysis and interpretation of data and drafting of the manuscript. HY participated in acquisition of data. YTan participated in critical revision of the manuscript for important intellectual contents. TT and AS participated in sample collection. JT participated in critical revision of the manuscript for important intellectual contents and obtaining fund. HO participated in study concept and design, interpretation of data, obtained funding, administrative and study supervision. All authors contributed to the article and approved the submitted version.
